# Lymphocyte transformation assay for C neoformans antigen is not reliable for detecting cellular impairment in patients with Neurocryptococcosis

**DOI:** 10.1186/1471-2334-12-278

**Published:** 2012-10-30

**Authors:** Katya C Rocha, Cinthia Pinhal, Sônia Cavalcanti, Monica SM Vidal, Matheus Toscano, Dewton Moraes-Vasconcelos, Alberto JS Duarte, Fernando LA Fonseca, Luiz Carlos de Abreu, Vitor E Valenti, Anete SG Grumach

**Affiliations:** 1 Laboratório de Dermatologia and Immunodeficiências, Departmento de Dermatologia, Faculdade de Medicina, Universidade de São Paulo. Av. Dr. Arnaldo, 455. 01246903, São Paulo, SP, Brazil; 2 Departamento de Morfologia e Fisiologia, Faculdade de Medicina do ABC. Av. Príncipe de Gales, 821. 09060-650, Santo André, SP, Brazil; 3 Departamento de Ciências Biológicas, Universidade Federal de São Paulo, Rua Prof. Artur Riedel, s/n, Diadema, SP, Brazil; 4 Departamento de Fonoaudiologia, Faculdade de Filosofia e Ciências, Universidade Estadual Paulista, UNESP. Av. Higyno Muzzi Filho, 737. 17.525-900, Marília, SP, Brazil; 5 Departamento de Patologia, Faculdade de Medicina do ABC. Av. Príncipe de Gales, 821. 09060-650 Santo André, SP, Brazil Av. Príncipe de Gales, 821. 09060-650, Santo André, SP, Brazil

**Keywords:** Lymphocytes, Antigens, Biases, Statistical, Cryptococcus, Methods, Cryptococcus neoformans

## Abstract

**Background:**

*Cryptococcus neoformans* causes meningitis and disseminated infection in healthy individuals, but more commonly in hosts with defective immune responses. Cell-mediated immunity is an important component of the immune response to a great variety of infections, including yeast infections. We aimed to evaluate a specific lymphocyte transformation assay to *Cryptococcus neoformans* in order to identify immunodeficiency associated to neurocryptococcosis (NCC) as primary cause of the mycosis.

**Methods:**

Healthy volunteers, poultry growers, and HIV-seronegative patients with neurocryptococcosis were tested for cellular immune response. Cryptococcal meningitis was diagnosed by India ink staining of cerebrospinal fluid and cryptococcal antigen test (Immunomycol-Inc, SP, Brazil). Isolated peripheral blood mononuclear cells were stimulated with *C. neoformans* antigen, *C. albicans* antigen, and pokeweed mitogen. The amount of ^3^H-thymidine incorporated was assessed, and the results were expressed as stimulation index (SI) and log SI, sensitivity, specificity, and cut-off value (receiver operating characteristics curve). We applied unpaired Student t tests to compare data and considered significant differences for p<0.05.

**Results:**

The lymphotoxin alpha showed a low capacity with all the stimuli for classifying patients as responders and non-responders. Lymphotoxin alpha stimulated by heated-killed antigen from patients with neurocryptococcosis was not affected by TCD4+ cell count, and the intensity of response did not correlate with the clinical evolution of neurocryptococcosis.

**Conclusion:**

Response to lymphocyte transformation assay should be analyzed based on a normal range and using more than one stimulator. The use of a cut-off value to classify patients with neurocryptococcosis is inadequate. Statistical analysis should be based on the log transformation of SI. A more purified antigen for evaluating specific response to *C. neoformans* is needed.

## Background

*Cryptococcus neoformans* causes meningitis and disseminated infection in healthy individuals, but more commonly in hosts with defective immune responses [1-3]. Cell-mediated immunity is an important component of the immune response to a great variety of infections, including yeast infections. Hence, impaired T-cell activation can increase the susceptibility to fungal pathogens like *C. neoformans*[4-6]. Graybill and Alford (1974) [7] observed reduced delayed-type hypersensitivity reactions against fungi like *Histoplasma capsulatum* and *Candida albicans,* as well as against a mitogen, in HIVseronegative patients recovered from cryptococcal disease, suggesting a decreased cellular response in these patients.

Lymphocyte transformation assay potentially offers a sensitive indicator for several conditions involving lymphocyte dysfunction due, for instance, to immunologic deficiency [8]. A decreased T-cell proliferative response to a mitogen, such as pokeweed (POKEWEED MITOGEN) or phytohemagglutinin (PHA), or to recall antigens, such as *C. albicans* or tetanus toxoid, could be an early marker of immune dysfunction in HIV-infected patients [9]. Additionally, lymphotoxin alpha could also be useful for evaluating sensitization to metals [10] in allergic diseases, for testing vaccine efficacy [11], and for assessing the effect of new immunostimulatory drugs [12].

There is, however, no general agreement among investigators on how to express data and to define, considering methodological variations, depressed or activated T-cell responses [13-17].

The clinical utility of lymphotoxin alpha depends on the skills of the clinical immunologist in interpreting data and on the establishment by the laboratory of a normal range [18]. In assays with ^3^H-thymidine incorporation as a marker of cell proliferation, the most usual way to represent the results is to calculate the difference between counts per minute (cpm) of the stimulated cells and the cpm of the non-stimulated cells (delymphotoxin alpha cpm). Also, the stimulation index (SI), the ratio of stimulated cells cpm and nonstimulated cells cpm, has been used.

This study was undertaken to evaluate a specific lymphocyte transformation assay to *Cryptococcus neoformans* in order to identify immunodeficiency associated to NCC as primary cause of the mycosis.

## Methods

The study was conducted between August 2004 and December 2006 and comprised 3 groups: a) 45 healthy volunteers; b) 24 poultry growers from Jumirim, SP, Brazil, who were individuals occupationally exposed to *C. neoformans*; and c) 25 HIV-seronegative patients with NCC. All patients with NCC were tested during their first or second episode of cryptococcal meningitis. Informed consent was obtained regarding blood samples from patients and control subjects, consent was obtained from parents for samples taken from babies. Patients receiving immunosuppressive medication or previously defined as immunodeficient and presenting with tumors or other infectious diseases were excluded. Age, gender, and T-lymphocyte counts of each group are presented in Table 1. For controls, we used blood samples from healthy volunteers and from healthy newborns. Three cord blood samples were obtained immediately after birth from healthy babies born at Maternal, Amparo, Brazil. All work procedures were approved by the Ethics Committee in Research of Instituto Emílio Ribas (protocol number 246/08) and followed Resolution 196/96 of the National Health Council of October 10, 1996.

**Table 1:** **Characterization of studied groups: number of studied individuals, median age, sex distribution, median of total lymphocytes, LTCD4**^**+ **^**and LTCD8**^**+**^

**Group**	**n**	**Lymphocyte cell/mL**	**LTCD4**^**+**^**cell/mL**	**LTCD8**^**+**^**cell/mL**	**Age (years)**	**Sex distribution**	
						**male**	**female**
Control individuals	45	1800 (1300–4390)	959 (556–2577)	497 (249–725)	26.5 (15–48)	26	19
Poultry growers	24	1720 (1180–3500)	828 (495–1380)	398 (167–763)	50.5 (15–75)	10	14
NCC patients	25	1580 (700–4390)	668 (15–1808)	459 (113–1137)	31.7 (15–60)	19	6

### Cryptococcal meningitis diagnosis

Cryptococcal meningitis was diagnosed by India ink staining of cerebrospinal fluid and cryptococcal antigen test (Immunomycol-Inc, SP, Brazil). *Cryptococcus neoformans* was identified by glossy, mucoid, and cream-colored colonies [19] cultured on Agar Niger and Agar Sabouraud (3 days at room temperature). Species confirmation was performed by positive urease test [19]. The protocol was approved by the Institutional Review Board of “Universidade de São Paulo” Medical School and of “Instituto Emílio Ribas.”

### Procedures

Lymphocyte transformation assay stimulated by POKEWEED MITOGEN, CMA, and *C. neoformans* antigen was established in all groups, according to the following steps: **Preparation of antigens:** Noncapsulated cells of *C. neoformans* serogroups A, B, C, and D were cultured separately on Sabouraud dextrose agar at 25°C for 3 days. Cells were harvested, mixed, and used to prepare heat-killed antigen (KAg) and soluble cell wall antigen (SAg). In order to prepare SAg, mixed yeasts were disrupted with glass powder, the final pellet was re-suspended in PBS 0.1 M (pH 7.2), and 10 mM protease inhibitor (methyl-phenyl-sulphonyl fluoride) was added. Yeast mixtures were incubated at 4°C for 48 hours. The suspension was centrifuged 3 times at 8000 rpm for 20 minutes. Supernatant was stored at −20°C until use. Protein concentration was determined by the Biuret method (protein concentration of pure antigen = 420 μg/mL). Heated-killed antigen mixed yeasts were suspended in PBS 0.1 M (pH 7.2) and heated at 121°C for 15 minutes. Cell concentrations were determined by hemocytometer (180 x 10^6^ yeasts/mL).

#### Lymphocyte Transformation Assay

Peripheral blood mononuclear cell (PBMC) from healthy volunteers and newborn cord blood cells were isolated by Ficoll-Hypaque density gradient (20 mL). The cells were centrifuged, washed 3 times at 4°C in NaCl (0.85%), re-suspended in RPMI 1640 medium, and supplemented with 10% AB human serum. The cellular concentration was adjusted to 2 × 10^6^ cells/mL. Triplicates of each of the following stimulators were used: pokeweed mitogen as positive control, and Candida metabolic antigen (CMA) as heterologous antigen. A triplicate of wells without stimulation was used as a negative control. The cells were then incubated in 96-well microplates in a 5% CO_2_ atmosphere at 37°C for 6 days. Eighteen hours before the final evaluation, 20 μL of ^3^H-thymidine were added to each well (1 μCi). On the sixth day, cultures were harvested on a glass fiber filter with a cell harvester device and prepared for counting. The amount of ^3^H-thymidine incorporated into DNA was assessed by liquid scintillation (Wallac Beta Plate equipment). Results were calculated as stimulation index (SI: mean of antigen-stimulated cells cpm divided by non-stimulated cells cpm). Log SI Cord blood cells were evaluated in order to test the mitogenic effect of the antigens.

PBMC (30 mL) from all groups were isolated, and the lymphotoxin alpha was performed as described previously. The following stimulators were used: KAg (4.5 ×10^6^ yeasts/mL), pokeweed mitogen (5 μg/mL), and CMA (5 μg/mL). In order to classify patients as responders and non-responders to stimulators, we applied an ROC (receiver operating characteristics) curve to establish a cut-off value and the 5^th^ to 95^th^ percentile range (as the normal range). We also calculated the specificity and sensitivity of lymphotoxin alpha using Win Episcope 2.0 software.

### Statistical analysis

Medians were compared, and unpaired Student *t* tests using a statistical software program (GraphPad Prisma 3.0) were used to analyze the lymphotoxin alpha. A *P* value less than 0.05 was applied to define a significant difference. Pearson’s coefficient was used to evaluate the correlation between lymphotoxin alpha stimulated by CMA and KAg for all groups studied. The 5^th^ to 95^th^ percentile range of lymphotoxin alpha was obtained by Sigma Stat software**.**

## Results

### Standardization of lymphocyte transformation assay to *C. neoformans* antigen

**Figure 1:**
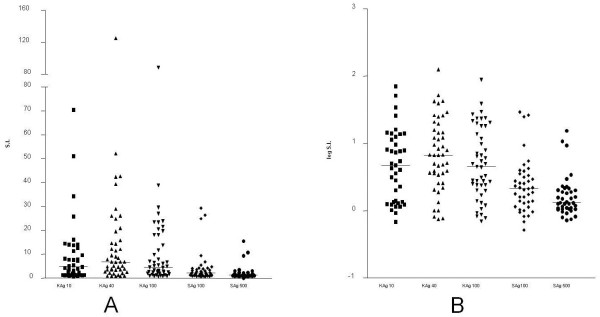
**Standardization of lymphoproliferative response to *C. neoformans *antigens (KAg and SAg) from healthy individuals (n = 45).** Results were calculated as: **A**) Stimulation index and **B**) log SI.

In an initial experiment (data not shown), we investigated the immunogenicity of SAg and KAg. For this purpose, PBMC from 3 individuals (healthy volunteers) and 2 HIV-seronegative patients with NCC were stimulated in vitro with several concentrations of SAg [4.2 μg/mL (dil. 1/100), 0.84 μg/mL (dil. 1/500), 0.42 μg/mL (dil. 1/1.000), 0.084 μg/mL (dil. 1/5000), 0.042 μg/mL (dil. 1/10000**)**] and KAg [18 x10^6^ yeasts/mL (dil. 1/10), 1.8 x10^6^ yeasts/ mL (dil. 1/100), 0.36 x10^6^ yeasts/ mL (dil. 1/500), 0.180 x10^6^ yeasts/mL (dil. 1/1000), 0.018 x 10^6^ yeasts/mL (dil. 1/10000)]. Both antigens were immunogenic, and the best responses were obtained with 4.2 μg/mL and 0.84 μg/mL for SAg, and 18 x10^6^ yeasts/mL (dil. 1/10) and 1.8 x10^6^ yeasts/mL (dil. 1/100) for KAg. Heated-killed antigen was more immunogenic than SAg, regardless of the applied concentration or the method used to evaluate the results (Figure 1). The lympho-proliferations detected with KAg dilutions 1/10, 1/40, and 1/100 were similar. A 1/100 dilution of SAg produced higher lymphocyte stimulation than a dilution of 1/500, and SAg elicited less lymphocyte proliferation than any concentration assayed of KAg (Figure 1). Data were evaluated as raw cpm and Δ cpm (data not shown), and there was no significant difference in SI of the results, even after logarithmic transformation (Figure 1). Table 2 shows P values from statistical analysis of lymphotoxin alpha obtained after stimulation by KAg and SAg of PBMC from the control group (Mann–Whitney test). 

**Table 2:** ***P *values from statistical analysis of lymphotoxin alpha obtained after stimulation by KAg and SAg of PBMC from the control group (Mann–Whitney test)**

	**KAg 1/10**		**KAg 1/40**		**KAg 1/100**	
	**SI**	**log SI**	**SI**	**log SI**	**SI**	**log SI**
**SAg 1/100**	.01	.009	.001	.001	.01	.003
**SAg 1/500**	.001	.001	.001	.001	.001	.001

### Determination of receiver operating characteristics (ROC) curve, sensitivity, specificity, cut-off value, and 5^th^ to 95^th^ percentile range of the lymphotoxin alpha

We analyzed the capability of the lymphotoxin alpha to classify patients with NCC as responders and non-responders by means of the cut-off value established by an ROC curve and the normal range (5–95 percentile**)**. The lymphotoxin alpha showed a low capacity with all the stimuli for classifying patients as responders and non-responders. Maximum area obtained from ROC curve was 53% for CMA (SI). The maximum sensitivity (96%) was observed with pokeweed mitogen stimulation, and the maximum specificity (82%) was obtained with KAg stimulation. The lymphotoxin alpha performed with CMA showed lower sensitivity and specificity compared with pokeweed mitogen or KAg. The cut-off value (SI) obtained from the ROC curve varied for different stimuli: 15 (KAg), 23 (pokeweed mitogen), and 12 (CMA). Applying the established cut-off value, patients with NCC classified as responders were: a) 10/25 (KAg), b) 8/25 (CMA), and c) 24/25 (pokeweed mitogen).

The lymphotoxin alpha on cells from patients with NCC was also analyzed compared with the 5th to 95th percentile range from a healthy volunteer population (normal range: CMA > 3.4 SI; KAg > 0.8 SI, and pokeweed mitogen > 8.4 SI. Patients with NCC considered responders were: a) 25/25 (KAg), b) 12/25 (CMA), and c) 25/25 (pokeweed mitogen).

### Lymphocyte transformation assay stimulated with fungal antigen and mitogen

**Figure 2:**
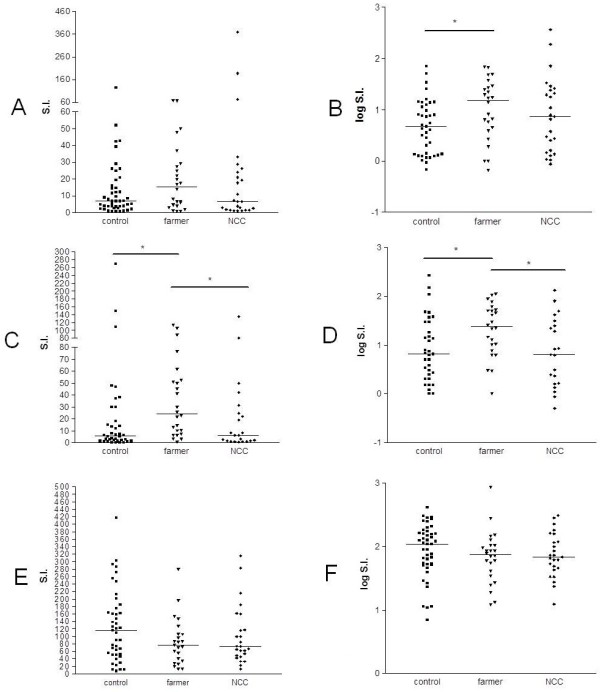
**Lymphoproliferative response to *C. neoformans *antigen (A and B) to *Candida albicans *antigen (C and D) and to pokeweed mitogen (E and F) from control group (nonoccupational exposure individuals, n = 45), poultry growers (occupational exposure individuals, n = 24) and neurocryptococcosis (NCC) patients (n = 22).** Results were expressed as: stimulation index (SI) (**A**, **C** and **E**) and log SI (**B**, **D** and **F**)

There was no significant difference between poultry growers and controls in specific T-lymphocyte response to KAg for values calculated as delymphotoxin alpha cpm (data not shown) or SI (*P* = 0.19). However, a significant difference was found in poultry growers compared with controls when data for lymphocyte proliferation was expressed as log SI (*P* = .03) (Figure 2). Poultry growers also showed higher specific lymphocyte response to *C. albicans* than controls (*P* = 0.004) (Figure 2). Both groups presented similar responses to pokeweed mitogen (*P* = 0.13) (Figure 2).

There was no correlation between lymphotoxin alpha results for different fungal antigens (KAg versus CMA) for the control group, regardless of how data were calculated (*r* =0.01 for delymphotoxin alpha cpm and *r* = −0.13 for SI). However, for poultry growers, there was a stronger correlation of the lymphotoxin alpha results for different fungal antigens when data were calculated as delymphotoxin alpha cpm and SI (*r* = 0.81).

Specific lymphocyte responses to cryptococcal antigen (KAg) by PBMC from patients with NCC, expressed by SI, log SI, or delymphotoxin alpha cpm (data not shown), were similar (*P* > .5) to responses by controls and poultry growers (Figure 2). Specific lymphocyte response to *C albicans* antigen (Figure 2) was lower when PBMC from patients with NCC and controls compared with poultry growers. Patients with NCC, farmers, and controls had similar responses (without statistical difference) to pokeweed mitogen (Figure 2). For lymphotoxin alpha of PBMC from patients with NCC, there was a medium correlation of CMA and KAg when data were calculated as SI (*r* = 0.7).

## Discussion

Clinical observations suggest that intact cellular immune response is a major determinant in the outcome of infection with *C. neoformans*[20,21]. Other immune mechanisms like antibodies to *Cryptococcus* antigens [22], antibody-dependent cell cytotoxicity [23], complement [24-27], and natural killer cells [27] also have a role in eliminating organisms. However, the frequency and severity of neurocryptococcosis and disseminated infection caused by *C. neoformans* in patients with AIDS clearly emphasizes the requirement for a T-cell–mediated immune response to this fungus [28,29].

An adequate antigen capable of eliciting lymphocyte activation in vitro and available for investigation of *C. neoformans–*specific T-cell response has not yet been identified; nor has the best approach for interpreting results been defined. The classification of a patient as responder or non-responder to an antigen could be useful to the clinical immunologist for classifying patients as immunocompetent or immunodeficient. We have studied lymphoproliferation to two different antigens: soluble cell wall antigen (SAg) and heat-killed yeast antigen (KAg). Since the capsular polysaccharide itself, a known virulence factor present in the intact *C. neoformans,* can suppress the lymphocyte proliferation [30], we prepared antigens with non-encapsulated yeasts. Both tested antigens were immunogenic to PBMC from healthy volunteers, but heat-killed yeast (KAg) was more immunogenic than soluble cell wall antigen (SAg).

Since Mody et al. (1999) [31] observed a mitogenic effect of cell wall antigen from *C. neoformans* to PBMC, we investigated whether the observed lymphocyte stimulation could be nonspecific (mitogenic effect). For this purpose, PBMC from cord blood were stimulated with both antigens (data not shown). Neither KAg nor SAg elicited stimulation of the cord blood cells. Therefore, the lymphoproliferation from healthy volunteers could be specific to *C. neoformans*.

Our results were similar to those observed by Miller and Puck (1984) [13], demonstrating that normal individuals (volunteers) without continuous exposure to *C. neoformans* had significant specific lymphoproliferative response to fungal antigen. Since *C. neoformans* is common in the environment [32], all individuals, even the healthy ones, could be exposed to the fungus. As reported, the volunteers not occupationally exposed are also responsive to fungal antigens. The environment is the only contaminating source, which exposed them daily (subjects exposed to occupational contact were excluded).

Based on the Miller and Puck study [13] and according to our findings, *Cryptococo* exposure occurs in the environment regardless of occupational exposure. In our study we pointed out that newborns do not respond to fungus and that the antigen used in the assays does not provide mitogenic effect. Therefore, subjects with no occupational exposure that respond to the fungi are related to a consequence of environmental exposure, as suggested by Miller and Puck [13].

Since contaminated feces and pigeon droppings are major sources of natural exposure to *C. neoformans*[33,34], we investigated the effect of this continuous exposure to specific lymphocyte response to *C. neoformans* antigen among poultry growers. They presented an increased lymphocyte response to *C. neoformans* antigen compared with non-exposed volunteers. It is interesting to note that this greater response could be only observed when data were analyzed after logarithmic transformation of stimulation index (log SI). When the data were calculated as delta cpm (data not shown) or SI, this statistical difference was not observed.

It is interesting to note that lymphoproliferative response to CMA antigen of peripheral blood mononuclear cell from poultry growers was higher than controls, regardless of the method used for data calculation. A possible explanation is that poultry growers are continuously exposed to *C. neoformans* (more than 10 years), increasing sensitized lymphocytes, which cross-react with *C albicans* antigen. Pietrella et al. (2002) [35] showed that immunized mice with mannoprotein from *C. neoformans* developed delayed-type hypersensitivity (DTH), a Th1 response, against lethal challenge with mannoprotein from *C. albicans*. As a matter of fact, there was a stronger and a moderate correlation (*r* = 0.81 and *r* = 0.7, respectively) of lymphoproliferative response by PBMC stimulated with CMA and KAg from poultry growers and patients with NCC, while PBMC from controls showed a poor correlation. Our data, as demonstrated by Pietrella et al. (2002) [35], also suggest a cross-reaction between epitopes of *C albicans* and *C. neoformans* in the T-cell response. On the other hand, the elevated lymphoproliferative response from poultry growers could be interpreted as a simple consequence of a better capability to respond or even a greater clonal expansion determined by continuously exposure to fungus.

In order to identify nonresponsive individuals or impairment of specific in vitro response to *C. neoformans*, the assay was also applied to HIV-seronegative patients with NCC. The patients’ specific lymphocyte response to KAg and pokeweed mitogen was not different from that observed with other groups (poultry growers or healthy volunteers), but it was lower for CMA, when compared with poultry growers. Our results were in accordance with those obtained by Levitz and North (1997) [36], who demonstrated that kinetics and magnitude of cellular immune response to *C. neoformans* antigen from patients and control group (without continuous exposure) are quite similar.

If we consider, as standardized in our laboratory, the 5^th^ percentile of SI as the normal range cut-off value for classifying individuals as responders or nonresponders to the antigens (KAg and mitogen), 100% of patients could be considered responders, but only 50% were responsive to CMA. On the other hand, if we use the ROC analysis to obtain a cut-off value, the number of responders and nonresponders is quite different, mainly because the cut-off values were much higher than the 5^th^ percentile of S.I. The ROC area, whose value represents the capability of the assay to discriminate subjects into two categories, showed us that the lymphotoxin alpha to *C. neoformans* antigen (like KAg) is a method that should not be used for diagnostic purposes, since this kind of method should present a ROC area of around 100%, high specificity, and high sensitivity.

Lymphocyte transformation assay stimulated by KAg from patients with NCC was not affected by TCD4+ cell count, and the intensity of response did not correlate with the clinical evolution of neurocryptococcosis. Cured patients without neurological sequelae presented a SI to KAg varying from 0.87 to 71, while patients who developed neurological sequelae or died presented high levels of specific lymphocyte response to KAg (SI, 1.27 - 368).

It might be expected that responses to short, relatively simple antigens such as synthetic peptides might indicate more precisely the difference between responders and nonresponders than the responses to complex antigens, since the potential number of epitopes and of responding cell clones would be much lower [33]. In addition, our data have been taken from a study of an uncommon disease caused by a fungus to which a great proportion of the population has been exposed.

It is important to mention that the diagnosis applied to the NCC patients is only directed to the diagnosis of fungal disease [37]. Although many *Cryptococcosis* cases are still diagnosed in immunocompetent individuals [38] the clinical management of patients with NCC is that cryptococcosis is an opportunistic disease. The evaluation of primary immunodeficiency after diagnosis is not indicated, it is not mentioned other immunological techniques (serological or cellular) to a diagnosis of the disease and its association with immunodeficiency.

Our results led us to conclude that specific lymphoproliferative response should be presented as SI, rather than raw cpm or delta cpm. Nevertheless, the comparison between two groups should be done by SI log transformation, which decreases variance. The use of mitogen stimulation reflects, with high sensitivity, nonspecific T-cell response capability, which was positive in all patients with NCC. The evaluated patients may not be classified as non-responders to C. neoformans by lymphoproliferative response assay. Considering all the results, lymphoproliferation assay applied to C. neoformans associated immunodeficiency diagnoses should be considered as a screening assay only. Furthermore, neurocryptococcosis is a disorder which has received large attention recently [39,40].

## Conclusions

Our data suggest that: 1) Analysis of lymphotoxin alpha response should be based on a normal range and analyzed using more than one stimulator; 2) The use of a cut-off value to classify patients with NCC as responders or non-responders to fungal antigens and mitogen is inadequate. We recommend the application of lymphotoxin alpha with another purified antigen to better evaluate a patient with NCC. It is advisable to use both stimuli to have a better overview of T-lymphocyte function.

## Abbreviations

HIV: Human immunodeficiency virus; NCC: Neurocryptococcosis; SAg: Soluble cell wall antigen; KAg: Killed antigen; ROC: Receiver operating characteristics; Δ cpm: Delymphotoxin alpha cpm; cpm: Counts per minute; SI: Stimulation index; PBS: Phosphate buffer solution; CMA: Candida metabolic antigen; PHA: Phytohemmaglutinin; PBMC: Peripheral blood mononuclear cells.

## Competing interests

We declare no conflict of interest.

## Authors’ contributions

All authors participated in the acquisition of data and revision of the manuscript. All authors conceived of the study, determined the design, performed the statistical analysis, interpreted the data and drafted the manuscript. All authors read and gave final approval for the version submitted for publication.

## Pre-publication history

The pre-publication history for this paper can be accessed here:

http://www.biomedcentral.com/1471-2334/12/278/prepub
